# Enhancing Antioxidant and Storage Stability of Upcycled Fruit Bars Through NADES-Based Lavender Extract Addition

**DOI:** 10.3390/molecules31152608

**Published:** 2026-07-27

**Authors:** Maja Benković, Manuela Panić, Marina Cvjetko Bubalo, Anja Damjanović, Ana Jurinjak Tušek, Davor Valinger, Jasenka Gajdoš Kljusurić, Tamara Jurina, Kristina Radošević, Ivana Radojčić Redovniković

**Affiliations:** 1 Faculty of Food Technology and Biotechnology, University of Zagreb, Pierrotijeva 6, 10000 Zagreb, Croatia; mcvjetko@pbf.hr (M.C.B.); adamjanovic@pbf.hr (A.D.); atusek@pbf.hr (A.J.T.); dvalinger@pbf.hr (D.V.); jgajdos@pbf.hr (J.G.K.); tamara.jurina@pbf.unizg.hr (T.J.); krado@pbf.hr (K.R.); irredovnikovic@pbf.hr (I.R.R.); 2 NADES Design Ltd., Borongajska c. 83 H, 10000 Zagreb, Croatia

**Keywords:** date fruit bars, fig fruit bars, grape pomace, lavender extract, natural deep eutectic solvents (NADESs)

## Abstract

This study investigated the development of upcycled functional fruit bars based on dates or figs and grape pomace, enriched with a lavender extract obtained using a novel multicomponent natural deep eutectic solvent (NADES). Supplementation with the NADES-based lavender extract significantly increased the total polyphenol content and antioxidant capacity, while improving the retention of total polyphenols during storage by approximately 10.3% in date-based bars and 2.8% in fig-based bars. The NADES-based lavender extract also reduced the percentage of yeast- and mold-positive samples from 60% to 20% in date-based bars after 30 days of storage at 22 °C. After 28 days of storage, supplemented bars showed better preservation of key sensory attributes, including appearance, color, odor, sweetness, bitterness and aftertaste. The stabilizing effects were more pronounced in date-based than in fig-based formulations, highlighting the influence of the food matrix on functional ingredient performance. Overall, the results indicate that NADES-based lavender extract is a promising functional ingredient for improving the physicochemical, sensory, and microbiological stability of upcycled fruit bars, particularly in date-based formulations.

## 1. Introduction

In recent decades, changes in lifestyle and socio-economic conditions have led to a growing demand for convenient and ready-to-eat food products [1]. At the same time, increasing consumer awareness of the relationship between diet and health has driven interest in nutritious and functional foods, particularly those that can be consumed on the go. Among such products, fruit bars have emerged as a popular option, as they combine convenience with a favorable nutritional profile. Compared to fresh fruits, fruit bars provide concentrated nutrients, extended shelf life, and enhanced convenience, making them a viable alternative [2,3].

Fruit bars are typically made from dried fruit, obtained through dehydration processes that reduce moisture content and extend shelf life while preserving nutritional value. However, drying can significantly affect the physicochemical and sensory properties of the final product, making it a critical factor in product development [4].

Beyond dried fruits, modern nutritional bars, including fruit, fruit–protein, and functional bars, incorporate a variety of ingredients. These often include sugar, glucose syrup, salt (sodium chloride), vitamins like ascorbic acid (vitamin C), and various food additives. Common additives include sugar substitutes like polyols (e.g., maltitol), stevia (*Stevia rebaudiana* Bertoni) glycosides, food acids such as citric acid, and hydrocolloids like sodium alginate, sodium carboxymethylcellulose, acacia gum, and pectin. Additionally, they may contain food colors and synthetic or natural flavors.

The potential of seed-free grape (*Vitis vinifera* L.) pomace as a valuable food ingredient and a source of important nutrients has been already recognized [5]. Similarly, dried fruits, particularly date palm (*Phoenix dactylifera* L.) and fig (*Ficus carica* L.), are widely used in the functional bar industry [6,7]. A combination of date palm, fig, and grape pomace is a recognized method for enriching these bars with phenolic antioxidants [8]. Development of upcycled fig- or date-based bars containing grape pomace skin as a functional ingredient has previously been described in [9]. The basic composition of the bar was optimized by the study, and the authors concluded that grape pomace can be used as a functional ingredient in the development of fruit bars. However, the authors also concluded that several issues remained to be addressed, particularly the shelf-life stability and the texture of the formulations.

Medicinal plant extracts are rich in bioactive compounds, particularly phenolics, which can enhance food shelf life, act as functional ingredients [10], or be incorporated into active packaging [11]. Lavender (*Lavandula officinalis* L.), alongside rosemary, sage, and thyme, possesses antioxidant, anti-inflammatory, and analgesic properties, making it a promising functional ingredient [12]. Lavender has a long history of use as a culinary herb and flavoring in foods, and it is not regarded as a novel food under Regulation (EU) 2015/2283 [13]. Nevertheless, as with other botanical extracts, the safety of concentrated preparations depends on their composition and intended level of use.

However, the use of plant extracts is limited by solvent choice, which affects extraction efficiency, bioactive stability, and sensory properties. For example, ethanol and methanol yield high phenolic contents but may introduce off-flavors or require removal steps, while water is safer but less efficient. Strong aromas or bitterness can also impact sensory acceptability, and phenolic compounds are prone to degradation from light, oxygen, temperature, and pH [14,15].

Natural deep eutectic solvents (NADESs) have recently emerged as a promising solution and a novel approach in the development of sustainable extraction and formulation strategies [16,17]. Beyond serving as efficient solvents, NADESs introduce a new conceptual framework in which the solvent itself can be designed as a functional component of the final extract. NADESs have proven to be excellent solvents for dissolving a wide range of compounds—both polar and non-polar—and are simple to produce, safe to handle, and highly adaptable for the selective extraction of targeted molecules [16]. Recent advances in green extraction highlight the use of natural deep eutectic solvents (NADESs) for the sustainable, selective, and efficient recovery of bioactive compounds from plant materials and food by-products [18,19,20]. Other physicochemical properties, including the ratio and composition of NADES components, can likewise be tuned to enhance extraction efficiency. Because NADESs are safe for direct use, ready-to-use extracts can be produced without the need for solvent purification [21]. These extracts can be rationally designed to provide specific functional attributes, such as antioxidant capacity, preservative activity, and broader biological effects. Through careful formulation, NADESs function not only as solvents but also as active contributors to the biological and technological performance of the extract [22]. Furthermore, the flexibility of NADES composition enables the design of multicomponent solvents with tailored physicochemical properties, improving the stability, solubility, and bioavailability of incorporated bioactive compounds [23]. Another advantage of multicomponent NADESs is that they allow for precise inclusion of multiple bioactive compounds without exceeding daily limits, ensuring product safety and regulatory compliance in dietary supplements [22]. A multicomponent NADES was formulated to function not only as an efficient extraction medium but also as a carrier of bioactive compounds, while additionally enhancing the sensory properties and freshness of the final product. The NADES, composed of betaine, citric acid, D-sorbitol, sucrose, and ascorbic acid in a defined molar ratio, was designed to efficiently solubilize and protect lavender phenolic compounds while providing a multifunctional, antioxidant-rich matrix that improves the longevity and freshness of the final tile application. For instance, betaine is a naturally occurring compound found in foods like beets, spinach, and grains. In the context of food, betaine is valued for its role in promoting liver health, supporting metabolic functions, and aiding digestion. It is commonly included in functional foods and dietary supplements for its potential to enhance athletic performance and improve cellular hydration [24]. Citric acid is a natural organic acid from citrus fruits, valued in foods for its tart flavor and its ability to regulate acidity and preserve freshness. It enhances taste, stabilizes formulations, and supports product shelf life [25]. Sorbitol is a sugar alcohol occurring naturally in fruits, valued as a sugar substitute in food due to its low glycemic index. Known for its moisture-retaining properties, sorbitol is widely applied in personal care products, pharmaceuticals, and foods requiring hydration and smooth texture, like sugar-free and low-calorie foods [26]. Sucrose is a disaccharide of glucose and fructose, commonly known as table sugar. It imparts sweetness and viscosity in formulations, enhancing flavor and texture. In food formulations, it helps manage osmotic properties, sweetness, and stability, working in conjunction with ingredients like betaine to balance moisture and texture [27]. Ascorbic acid is a nutrient found in many fruits and vegetables, recognized for its strong antioxidant properties. In foods, it prevents oxidation, preserves color and flavor, and supports nutritional value in fortified products and supplements [28]. Furthermore, the molar ratios of the designed NADESs were formulated in accordance with regulatory requirements and guidelines for the daily intake of each NADES component in the final food product [29]. Therefore, multicomponent NADESs were developed to allow precise incorporation of multiple bioactive compounds without exceeding the recommended daily intake limits of individual components. For example, the European Food Safety Authority (EFSA NDA Panel (EFSA Panel on Nutrition, 2019 [29]) recommends a maximum daily dose of 1.5 g of betaine for adults in dietary supplements, ensuring safe consumption. In the case of sorbitol, according to Regulation (European Commission, 2012) No. 231/2012 [30] and standard product formulations, it is commonly used at levels such as 4370 mg per 5 mL in syrups, remaining well within safe limits for its intended use.

This study presents a novel strategy for developing upcycled functional fruit bars based on dates or figs and grape pomace, enriched with a lavender extract obtained using a purpose-designed multicomponent NADES. The developed NADES served as both an extraction medium and a carrier of bioactive compounds. The objective of the study was to evaluate the effects of the NADES-based lavender extract on the physicochemical, antioxidant, microbiological, sensory, and storage stability properties of the fruit bars. It was hypothesized that supplementation with the developed extract would improve the functional properties and storage stability of the final products.

## 2. Materials and Methods

### 2.1. Materials

The raw materials used in this study were dried fig, dried date, coconut flour, hazelnut, and cocoa powder with 10–12% fat, all provided by Nutrigold (Zagreb, Croatia); citrus peel powder, orange peel powder, and vanilla sugar, all provided by Arcadie (Méjannes-lés-Alés, France); and Graševina variety grape pomace from Požega, Slavonija County, harvest year 2021., supplied by Kutjevo d.o.o. (Kutjevo, Croatia). The basic characteristics of the pomace were 46.55% dry matter, 7.73% protein, 4.13% fats, 4.53% sugars, 29.13% fiber, and pH = 4.6. Prior to for the preparation of the bars, pomace was analyzed for the presence of pesticides, and a full microbiological safety profile was done that confirmed its adequacy for use in food products [9]. *Lavandula angustifolia* L. plants were purchased from Suban (Strmec Samoborski, Croatia), year of harvest 2021., originating from Croatia.

The chemicals and reagents used in this study were as follows: ethanol (Kefo d.o.o., Ljubljana, Slovenia), methanol (J.T.Baker, Hampton, NH, USA), Folin–Ciocâlteu reagent (Kemika, Zagreb, Croatia), 1,1-diphenyl-1-picrylhydrazyl (DPPH) (Sigma Aldrich, Darmstadt, Germany), gallic acid, 98% (AcrosOrganics, Fair Lawn, NJ, USA), sodium acetate trihydrate (CH3COONa·3H_2_O) (J.T.Baker, Deventer, the Netherlands), 2,4,6-triphenyl-1,3,5-triazine (TPTZ) (Sigma-Aldrich, Darmstadt, Germany), sodium carbonate (Na_2_CO_3_), p.a. (Gram Mol, Zagreb, Croatia), 6-hydroxy-2,5,7,8-tetramethylchroman-2-carboxylic acid (Trolox) (Fluka, Buchs, Switzerland), iron (III) chloride hexahydrate (FeCl_3_·6H_2_O) (GRAM-MOL d.o.o., Zagreb, Croatia), iron (II) sulfate heptahydrate (FeSO_4_·7H_2_O) (Sigma-Aldrich, Darmstadt, Germany), and acetic acid 99.5% (CH_3_COOH) (T.T.T. d.o.o., Sveta Nedjelja, Croatia). The chemicals used for the preparation of the NADES were betaine (Fagron, Bologna, Italy), citric acid (Fagron, Bologna, Italy), D-sorbitol (Fagron, Bologna, Italy), sucrose (Sladorana Županja d.d., Županja, Croatia), ascorbic acid (Fagron, Bologna, Italy), and purified water Ph.Eur. grade (Fagron Croatia d.o.o., Donja Zelina, Croatia).

### 2.2. Methods

#### 2.2.1. NADES and Lavender NADES-Based Extract Preparation

Betaine, citric acid, D-sorbitol, sucrose, and ascorbic acid, at a molar ratio of 1:0.2:1:1:1, along with 30% (*v*/*v*) purified water, were placed in a in a 500 mL reagent bottle with a screw cap and were then placed in an ES-20/60 Shaker–Incubator (Biosan, Riga, Latvia), stirred, and heated to 50 °C for 2 h until a homogeneous transparent colorless liquid was formed. The preparation resulted in 611.55 g of 30%-diluted NADES as an odorless and colorless viscous liquid. After the NADES was prepared, 50 g of dried lavender plant (Hvar, Croatia) was added to 250 g of diluted NADES, and the resulting mixture was gently stirred at 60–65 °C for 2 h (Biosan, Riga, Latvia). The obtained mixture was subjected to filtration, yielding the filter cake of the used herbal residue and 184.50 g (73.8%) of filtrate as a viscous, pale yellowish liquid.

#### 2.2.2. HPLC Analysis of Lavender NADES Extract

Polyphenols were identified and quantified using an HPLC system (1260 Infinity II, Agilent, Santa Clara, CA, USA) coupled with a diode array detector (UV/DAD, 1260 Infinity II, Agilent, USA) and an automatic sampler (1260 Infinity II, Agilent, USA). Separation was achieved on a Poroshell 120 SB C18 column (150 mm, 4.6 mm, 5 µm; Agilent, USA) using H_2_O with 0.25% AcOH (A) and ACN (B) as the mobile phases. Gradient elution was modified as follows: 0–7.5 min 10% B, 7.5–15 min from 10% to 15% B, and 15–25 min from 15% to 10% B. The flow rate was 1 mL min^−1^. The sample injection volume was 15 μL, and the samples were always filtered through 0.22 μm polytetrafluoroethylene (PTFE) filters prior to injection. The column temperature was kept at 40 °C. UV-DAD acquisitions were carried out in the 200–600 nm range, while chromatograms were acquired at 280, 320, and 340 nm.

The retention times and UV–Vis spectral characteristics of the identified polyphenolic compounds were confirmed by comparison with authentic external standards. Quercetin and luteolin-7-O-glucoside were quantified at 360 nm, whereas gallic acid, catechin, chlorogenic acid, rosmarinic acid, syringic acid, and trans-sinapic acid were measured at 280 nm, corresponding to their respective absorption maxima. Quantification of all detected polyphenols was performed using calibration curves of external standards at their wavelengths of maximum absorbance. All HPLC analyses were conducted in triplicate, and the results are expressed as micrograms of compound per gram of dry weight (dw). The HPLC-DAD method was validated by assessing linearity, correlation coefficient (R^2^), limit of detection (LOD), and limit of quantification (LOQ) using authentic external standards. Calibration curves were prepared over concentration ranges of 0.01–1.0 mg mL^−1^ for gallic acid, catechin, syringic acid, chlorogenic acid, trans-sinapic acid, and quercetin, 0.01–0.5 mg mL^−1^ for luteolin-7-O-glucoside, and 0.01–0.05 mg mL^−1^ for rosmarinic acid. Excellent linearity was obtained for all analytes (R^2^ = 0.99945–0.99999). The LOD and LOQ values ranged from 0.00067 to 0.036 mg mL^−1^ and from 0.002 to 0.110 mg mL^−1^, respectively, confirming the suitability of the method for quantitative analysis.

#### 2.2.3. Fruit Bar Preparation

First, the seeds and stalks were separated from the rest of the pomace using a laboratory-made sieve with a pore diameter of 5 mm. After separating the seeds, the obtained grape skin was blended (Delimano, Cadempino, Switzerland) to a mushy structure. Dried dates and dried figs were also blended to a mushy texture. The hazelnuts were ground using the same blender, and the ground hazelnuts were mixed with cocoa powder at a 1:1 ratio.

Fruit bars were prepared according to a composition mentioned in a previous study [9]. Two types of bars were prepared: fig-based and date-based. The basic fruit bar mixtures consisted primarily of dates or figs (64.5%, *w*/*w*), grape pomace (grape silver skin; 33.58%, *w*/*w*), and a cocoa/hazelnut mix (1.92%, *w*/*w*). Four separate batches of basic mixtures were prepared—two fig-based and two date-based. After the basic mixture batches were prepared, the batches were supplemented with an additional spice mix (orange/lemon/vanilla, added at 2 g per 100 g of basic mix). The fig-based basic mixture was additionally supplemented with 0.5 g per 100 g of vanilla powder. For each fruit bar type, one batch was considered a control batch, which contained no lavender NADES extract (sample D for date-based bar and sample F for fig-based bar), and the other batch contained 1% (*w*/*w*) lavender NADES extract (sample D + N for date-based bar and sample F + N for fig-based bar). Prior to selecting the final formulation, preliminary trials were conducted to evaluate the technological suitability of different levels of both NADES and NADES-based lavender extract in the fruit bar formulation (1, 5, and 10% (*w*/*w*)). Based on these preliminary findings, supplementation with 1% (*w*/*w*) NADES-based lavender extract was selected for the final experimental design and evaluated as a proof-of-concept formulation.

The bars were prepared by weighing and homogenization of the raw materials into a mush-like mixture. After mixing, the mixtures were weighed (20 g each), placed in a mold, and put in a convective oven (InkoLab ST60T, Zagreb, Croatia) to dry for 8 min at 180 °C. The resulting 20 g bars (30 bars per batch) were cooled, individually packed in PA/PE vacuum bags, sealed and placed in a refrigerator (+4 °C) for storage until further analysis.

#### 2.2.4. Moisture Content

The moisture content of the fruit bars was determined by the gravimetric drying method at a temperature of 105 °C to a constant mass. Measurements were conducted in duplicate, and the results were expressed as the mean value ± standard deviation (SD).

#### 2.2.5. Bar Extract Preparation for Physicochemical Analyses

Bar extracts were made using ethanol (70%) as a solvent. The extraction conditions (70% ethanol, sample-to-solvent ratio of 1:30 (*w*/*v*), extraction at 50 °C for 30 min) were selected according to the method described by Benković et al. [9], which has been validated for the extraction of phenolic compounds from fruit-based matrices. Three grams of the crushed sample was weighed and added to 90 mL of the extraction solvent previously heated to 50 °C in an oil bath (IKA HBR4 digital, IKA-Werke, Staufen, Germany). The extraction was carried out for 30 min at a mixer speed of 500 rpm in covered glass beakers, in order to prevent the evaporation of the solvent. The obtained extract was filtered on a vacuum filtration set (Rocker 300—LF 30, New Taipei City, Taiwan) and stored in 50 mL Falkon cuvettes. The samples were kept in a freezer until further analyses. Extracts were made and analyzed successively on days 0, 7, 14, 21, and 28 of storage. Ethanolic extraction was performed exclusively for analytical purposes to obtain standardized extracts suitable for the spectrophotometric determination of total polyphenol content (TPC), DPPH radical scavenging activity, and FRAP values, while the NADES-based lavender extract itself was used as the functional ingredient incorporated into the fruit bars.

#### 2.2.6. Total Dissolved Solids, Conductivity, and pH of the Extracts

Conductivity and the total dissolved solids were measured as complementary indicators of physicochemical stability during storage, reflecting changes in the soluble constituents of the fruit bar matrix. Conductivity and the total dissolved solids were determined using a conductometer (SevenCompact, MettlerToledo, Greifensee, Switzerland) by immersing the probe in the liquid extract. Three parallel measurements were carried out. The pH value of the extracts was determined using a pH meter (Jenco 601A, Schaumburg, IL, USA) by immersing the pH probe in the extract. Three parallel measurements were also carried out, and the results were expressed as the mean value ± SD.

#### 2.2.7. Determination of Total Polyphenolic Content (TPC)

The total polyphenolic content (TPC) of the prepared extracts was determined spectrophotometrically by the Folin–Ciocâlteu reagent, as described previously by Singleton and Rossi (1965) [31]. The measurements were carried out in triplicate and the results were expressed as mean values ± standard deviation of mg gallic acid equivalents (GAE) per gram of dry matter (mg GAE g dm^−1^).

#### 2.2.8. Determination of Antioxidant Activity (DPPH and FRAP Methods)

The antioxidant activity was determined spectrophotometrically using the DPPH (2,2-diphenyl-1-picrylhydrazyl) and the FRAP (Ferric Reducing Antioxidant Power) methods. The measurements were carried out in triplicate, and the results were expressed as mean values ± standard deviation of the molar fraction (mmol) of Trolox equivalents per gram of dry matter (mmol TE g dm^−1^) for the DPPH method, and as molar equivalents of FeSO_4_·7H_2_O per gram of dry matter (mmol FeSO_4_ g dm^−1^) for the FRAP method.

#### 2.2.9. Sensory Analysis of Fruit Bars

Sensory properties of the produced fruit bars were analyzed based on the hedonistic scale with grades from 1 to 5, with 1 indicating the least acceptability and 5 the highest acceptability. The properties that were analyzed were appearance, color, smell, sweetness, bitterness, aftertaste (defined as acceptability), and texture. Samples were presented to the analysts in a closed, marked package, in a random order, and the analysis was conducted on the day of production (0 day) and after 28 days of storage. The panel comprised 11 trained (prior screening for sensory acuity, standardizing terminology, and calibrating evaluations to identify specific organoleptic attributes and use scales to quantify intensities without bias) analysts, aged 22–45 years (3 male and 8 female). Each panelist signed an informed consent form, informing the panelist about the nature of the samples, the purpose of the study, possible allergies, and personal data protection. Also, special care was taken that each panelist who participated in the sensory analysis on day 0 also participated in the analysis on day 28, to ensure the integrity of the results. The relative stability of each tested sensory property was calculated according to Equation (1):

(1) $$Relative\;stability\left(\%\right)=\frac{P_{28}-P_{0}}{P_{0}}\times 100$$

where *P*_28_ represents the value of a given property after 28 days of storage and P_0_ represents the value of a given property at day 0. The results are presented as the mean value ± SD.

#### 2.2.10. Microbiological Stability of Fruit Bars

Microbiological analysis of the fruit bars included the presence of aerobic mesophilic bacteria, *Enterobacteriaceae*, *Staphylococcus aureus*, *Salmonella* spp., molds, and yeasts, all tested according to the valid microbiological norms and criteria in the Republic of Croatia (HRN EN ISO 4833-2:2013 [32], HRN ISO 21528-2:2017 [33], HRN EN ISO 6888-1:2021 [34], HRN ISO 21527-2:2012 [35] and HRN EN ISO 6579-1:2017 [36]). Stability was tested on days 0, 15, and 30 of storage, at 4 °C and 22 °C, each analysis with measurements on 5 samples, each originating from its own individual packaging. Microbiological analyses were performed by an accredited laboratory according to standardized ISO methods and the valid microbiological criteria in the Republic of Croatia. The laboratory determined microbial counts (CFU/g) and evaluated compliance with the prescribed microbiological criteria. For the purpose of comparing microbiological stability among formulations during storage, the results are presented as the percentage of positive samples (*n* = 5), while the laboratory detection limits were <100 CFU/g for aerobic mesophilic bacteria, <1 CFU/g for *Enterobacteriaceae* and coagulase-positive *Staphylococcus aureus*, and <10 CFU/g for yeasts and molds. The results are shown as percentages of samples that proved to be positive for each of the tested microorganisms on days 0, 15, or 30 of storage.

#### 2.2.11. Statistics and Data Analysis

Statistical analysis was performed using Statistica software v. 14.1. (StatSoft GmbH, Hamburg, Germany). First, the chi-squared normality test was performed, which showed that normality violation was present, and the subsequent analysis was adjusted accordingly. Further analysis included mixed-effects MANOVA, with composition (date/fig as a single categorical variable) and lavender NADES-based extract addition as categorical predictors and storage time at 5 levels (0, 7, 14, 21 and 28) as a within-effect factor. Since normality violation was present in the data, Pillai’s trace multivariate test was used to estimate the effects of composition, NADES-based lavender extract, and storage time, as well as their combination, on the dependent variables (moisture, TDS, conductivity, pH, TPC, DPPH, and FRAP). In the case of sensory properties, the same procedure was applied, with the exception of performing a univariate Pillai’s trace test, since the sensory properties were estimated at only 2 time points (0 and 28 days of storage). In sensory statistical analysis, composition (date/fig as a single categorical variable) and NADES-based lavender extract were considered as categorical predictors and storage time at 2 levels (0 and 28 days) as a within-effect factor, while appearance, color, smell, sweetness, bitterness, aftertaste (defined as acceptability), and texture were considered dependent variables. Post hoc comparisons included the Dunn–Bonferroni test with confidence limits of 95%, and a significance level of 0.05.

## 3. Results and Discussion

### 3.1. Lavender NADES-Based Extract Characterization

This manuscript presents a novel strategy for the development of functional, upcycled date- or fig- and grape-based fruit bars enriched with a lavender extract obtained using a purpose-designed NADES. Lavender (*Lavandula* spp.) was selected due to its rich phenolic profile and well-documented antioxidant potential. Previous studies have demonstrated that NADESs improve the extraction efficiency of phenolic and other bioactive compounds from plant matrices, thereby enhancing the functional potential of plant extracts [37,38,39].

The multicomponent NADES efficiently served as both an extraction medium and a carrier of bioactive compounds, contributing to the improved antioxidant, sensory, and stability-related properties observed in the developed fruit bars. In addition, the selected food-grade NADES components were formulated in accordance with regulatory recommendations. Considering the formulation used in this study, a standard 20 g fruit bar containing 1% (*w*/*w*) NADES extract provides approximately 19 mg of betaine and 30 mg of sorbitol per serving. This corresponds to only about 1.3% of the EFSA maximum recommended daily intake for betaine (1.5 g day^−1^), while the sorbitol content is negligible compared with amounts commonly associated with gastrointestinal effects. Therefore, consumption of the developed fruit bars remains well within established safety limits for these NADES components. Although the formulation was based on food-grade ingredients, and the estimated intake of the NADES components remained within regulatory limits, the safety of the final NADES-based extract should be further confirmed through in vitro cytotoxicity studies prior to commercialization.

The chemical composition of the resulting NADES–lavender extract was characterized using high-performance liquid chromatography (HPLC) (Table 1), enabling the identification and quantification of major phenolic acids and flavonoids. The NADES-based lavender extract contained eight identified phenolic compounds: catechin, quercetin, gallic acid, luteolin-7-O-glucoside, chlorogenic acid, rosmarinic acid, syringic acid, and trans-sinapic acid. Among these, rosmarinic acid (5.3 ± 0.4 µg g _dw_^−1^) was the predominant constituent, consistent with previous reports describing it as a major phenolic in *Lavandula* species. Although rosmarinic acid was the predominant identified phenolic compound, the sum of the phenolics quantified by HPLC accounted for only a fraction of the total phenolic content determined by the Folin–Ciocâlteu assay (15 mgGAE g^−1^). This difference was expected because the Folin–Ciocâlteu method measures the overall reducing capacity of the extract, including numerous unidentified phenolic constituents and other reducing compounds that were not individually quantified by the targeted HPLC analysis. In addition, blank NADES samples exhibited measurable responses in the Folin–Ciocâlteu, DPPH, and FRAP assays, indicating that the NADES itself contributed to the measured reducing capacity.

Gallic acid (0.3 ± 0.0 µg g _dw_^−1^) and chlorogenic acid (3.2 ± 0.0 µg g _dw_^−1^) were present in moderate amounts, indicating that the selected NADES formulation is effective in extracting low-molecular-weight phenolic acids. Quercetin (1.3 ± 0.2 µg g _dw_^−1^) and syringic acid (0.7 ± 0.9 µg g _dw_^−1^) were detected in lower amounts, consistent with their minor occurrence in Lavandula species. Trans-sinapic acid was also found in trace amounts. The particularly low levels of luteolin-7-O-glucoside align with observations that this flavonoid is typically present at minor concentrations in *L. angustifolia* [40].

Lavender extract demonstrated high polyphenol content (15 mg GAE g^−1^), outperforming most published extraction results. For example, in studies applying natural deep eutectic solvents (NADESs), two systems were used: choline chloride/formic acid (ChCl:FA) and choline chloride/ethylene glycol (ChCl:EG). Extracts obtained with ChCl:FA reached 9.76 mg GAE g^−1^ TPC, 20.83–104.39 mg TE g^−1^ antioxidant capacity, and 0.20 mg g^−1^ total individual phenolics, demonstrating that even the most efficient NADES system produced substantially lower phenolic yields compared with our extract, while ChCl:EG showed even weaker extraction efficiency [41]. These differences in the phenolic profile are likely attributable to differences in the NADES composition and extraction conditions, as well as variability in the lavender plant material (e.g., chemotype, cultivation, and harvest conditions). Since the extraction selectivity of NADESs strongly depends on their composition, different solvent systems preferentially extract different groups of phenolic compounds. Likewise, ethanol-based extracts of lavender by-products contained 16.08 mg GAE g^−1^ phenolics and 3.89 mg g^−1^ flavonoids, values comparable to those obtained with our method [42]. Additional research further confirms that although NADESs offer sustainable, selective extraction—particularly for phenolic acids such as rosmarinic and chicoric acid—total phenolic yields generally remain lower than those obtained using conventional solvents.

### 3.2. Physicochemical Properties and Stability of Fruit Bars

Prior to selecting the final supplementation level, preliminary formulation trials were conducted using both blank NADES and NADES-based lavender extract at concentrations of 1, 5, and 10% (*w*/*w*). These trials were performed to assess the technological suitability and sensory acceptability of the formulations. Based on the preliminary observations, 1% (*w*/*w*) NADES-based lavender extract was selected for the final experimental design, providing the most favorable balance between functionality and acceptability to the panelists. The blank NADES formulation was not included in the subsequent physicochemical, microbiological, or storage experiments, and the results presented in this study therefore refer exclusively to the combined NADES–lavender formulation. The prepared and characterized NADES-based lavender extract 1% (*w*/*w*) was subsequently incorporated into fruit bars formulated from dates or figs and grape pomace, supporting a sustainable upcycling approach. Furthermore, physical properties of the fruit bars upcycled by using lavender NADES extracts as a component were determined in terms of dry matter content, pH, TDS, and conductivity, and the results are shown in Table 2.

The prepared fruit bars, formulated from dates or figs and grape pomace, and further enriched with 1% (*w*/*w*) NADES-based lavender extract, were characterized for their key physicochemical properties. At the initial time point (day 0), the date bars exhibited a dry matter content of 64.36%, a pH of 5.55, total dissolved solids (TDS) of 61.83 mg L^−1^, and an electrical conductivity of 129.87 µS cm^−1^. Date bars with lavender NADES extract showed a dry matter content of 63.45%, pH of 5.49, TDS of 62.67 mg L^−1^, and conductivity of 119.30 µS cm^−1^. Fig bars displayed a dry matter content of 61.04%, pH of 5.59, TDS of 46.37 mg L^−1^, and conductivity of 93.97 µS cm^−1^, while fig bars with lavender NADES extract had a dry matter content of 64.13%, pH of 5.49, TDS of 52.47 mg L^−1^, and conductivity of 101.50 µS cm^−1^.

These measurements provide a baseline description of the fruit bars’ moisture, acidity, soluble solids, and ionic content, which are fundamental parameters for assessing product quality and stability.

**Figure 1:**
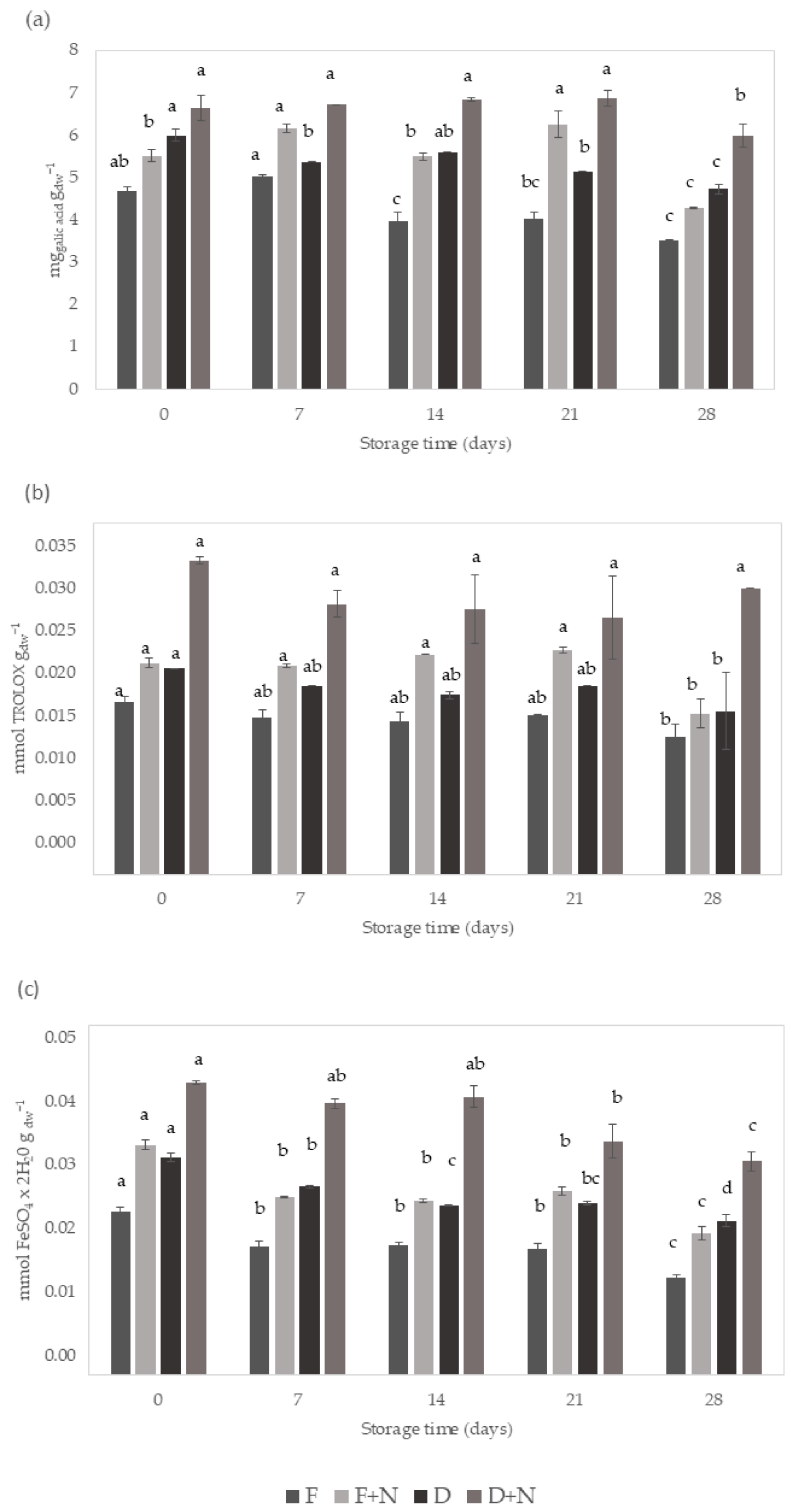
Changes in chemical properties of the fruit bars during 28 days of storage: (**a**) TPC; (**b**) antioxidant capacity determined by the DPPH method; (**c**) antioxidant capacity determined by the FRAP method. Data are presented as the mean ± standard deviation (*n* = 3). D—date fruit bar; D + N—lavender–NADES-supplemented date bar; F—fig fruit bar; F + N—lavender–NADES-supplemented fig bar. Different letters above the bars represent significant differences based on the Dunn–Bonferroni post hoc test at *p* < 0.05.

The chemical properties of the prepared fruit bars were evaluated by determining their total polyphenol content and antioxidant capacity using the DPPH and FRAP methods. Date bars showed the highest polyphenol content among the basic formulations, while fig bars contained slightly less. The increase in total polyphenol content was expected, as the NADES-based lavender extract itself contained a high concentration of phenolic compounds, which contributed directly to the overall polyphenol content of the enriched fruit bars (Figure 1).

Antioxidant capacity measured by the DPPH method indicated that fig bars had slightly lower activity compared to date bars. The incorporation of lavender NADES enhanced the antioxidant capacity, particularly in the date-based bars. A similar pattern was observed with the FRAP method: the enriched date bars demonstrated the highest antioxidant activity, whereas fig bars, regardless of supplementation, showed comparatively lower values (Figure 1).

The stability of the fruit bars was monitored over 28 days of vacuum-packed storage in plastic films, assessing physicochemical properties, antioxidant capacity, and microbiological stability to evaluate the effects of date, fig, and NADES lavender extract supplementation on product quality.

The TPC, DPPH, and FRAP results of the fruit bars are shown in Figure 1, while the multivariate Pillai’s test results demonstrating the effects of storage time, composition, and NADES-based lavender extract on the analyzed physical and chemical properties are shown in Table 3, and microbiological stability results are shown in Table 4.

As shown in Table 2, all fruit bar types generally maintained dry matter levels around 61–64% across the storage period, with small fluctuations. The pH values remained relatively stable, ranging from approximately 5.4 to 5.6 for all bars throughout the testing period, indicating limited acidification or spoilage. Total dissolved solids (TDS) values tended to increase over the storage period for most fruit bar types, suggesting additional solubilization or migration of solids from the matrix into the extract during storage. Conductivity values also typically rose throughout storage, which is consistent with increased ionic solutes dissolved in the bars [37,38,39].

In terms of specific observations by fruit bar type, date bars showed a slight decrease in dry matter after 28 days of storage, along with the lowest initial values of TDS and conductivity. These values started to rise after 14 days, likely reflecting diffusion processes or moisture migration. When lavender NADES extract was added to the fruit bars, some specific effects were noticed: dry matter and pH values remained stable, while TDS and conductivity values were larger in comparison to the non-supplemented bars and peaked at later stages, especially after day 14, indicating ongoing solute release or transformation. For fig fruit bars, similar stable dry matter and pH trends were detected. As for TDS and conductivity, in general, those values were lower for fig-based bars, which is in accordance with data from the literature [9]. However, the fig TDS and conductivity values started lower and showed an upward trend over time. When lavender NADES extract was added to the fruit bars, there was no significant rise in TDS and conductivity in comparison to the non-supplemented fig bars; however, there was a slight drop in pH value of the bars, connected to the sole composition of the NADES, which contained citric acid, contributing to the reduced pH. TDS and conductivity values for the lavender–NADES-supplemented fig-based bars both increased toward day 28 of storage. These patterns suggest that all types of bars retained their basic composition and safety (pH stability), while the observed increases in TDS and conductivity point to gradual changes in solubility and ionic strength, which are typical for stored fruit-based products. The lavender NADES bars tended to show slightly higher measures, possibly due to ingredients enhancing extractability or solute migration.

In general, the dry matter content of the bars dropped after 28 days, due to the incremental drying with prolonged storage. Changes in the dry matter content of lavender–NADES-supplemented bars were significantly lower than those of un-supplemented bars, indicating that the NADES-supplemented bars had higher stability in terms of moisture migration from the product matrix to the surrounding air. The change in the pH values of the supplemented bars was significantly greater than the change in the non-supplemented ones, which was connected to the higher contents of polyphenolic bioactive compounds in the extract, as well as their permeation throughout the fruit bar matrix during storage. TDS and conductivity values, which usually act as the first indicators of the presence of bioactives in extracts, showed an increase during storage in both supplemented and non-supplemented bars, although no clear effect of NADES-based lavender addition was detected on those properties. In comparison to the literature data, the pH values (around 5.4–5.6) and total dissolved solids (TDS 55–80 mg/L) obtained in this study for date fruit bars align with reports for date fruit juices and concentrates, indicating that these levels are typical in date products during processing or storage [43,44]. Furthermore, conductivity and dry matter values were also consistent with the literature data for date products [45]. Also, the reported pH (~5.5) and TDS values (46–70 mg/L) are comparable to those reported for fig pastes and fruit extracts, where pH, titratable acidity, and conductivity were measured under similar conditions [46].

To corroborate the above claims statistically, Pillai’s multivariate test was performed to estimate the individual effects of storage, composition, NADES-based lavender extract, and their combination on dry matter content, pH, TDS, and conductivity. As shown in Table 3, dry matter content was significantly influenced by storage time, as well as the combined effects of storage and composition, storage and NADES-based lavender extract, and all three combined. The same can be seen for TDS and conductivity, which were also significantly influenced by all effects. This indicates that storage stability of the fruit bars is a complex phenomenon, depending not solely on the storage time passed but also on the composition and the addition of lavender–NADES as a stabilizer. In the case of pH, no individual effects or their combinations showed any significant influence on pH values.

The stability of total phenolic content (TPC) after 28 days of storage differed among the fruit bar formulations. The D + N formulation showed the highest TPC stability (89.07%), whereas the F formulation exhibited the lowest stability (74.67%), indicating a greater reduction in phenolic compounds during storage. The D (78.76%) and F + N (77.53%) formulations displayed intermediate TPC stability (Table S1).

Overall, the incorporation of NADES-based lavender extract improved TPC stability, increasing it by approximately 2.8% in fig bars (F→ F + N) and by about 10.3% in date bars (D → D + N). The effect was more pronounced in the date-based formulation, suggesting that the influence of the NADES-based lavender extract depends on the composition of the fruit matrix. A possible explanation for the greater effect observed in the date-based bars is the difference in fruit matrix composition. Dates contain higher levels of soluble sugars, whereas figs are richer in dietary fiber, which may influence the interactions between the matrix and the NADES-derived phenolic compounds during storage [37,47]. Similar natural degradation of phenolics during storage was also shown in a study by Aslam et al. (2023) [48], who developed fruit bars containing roselle and fig and argued that the decrease in TPC was due to the presence of polyphenol oxidases. The NADES-supplemented bars showed higher TPC values than the non-supplemented ones and maintained elevated TPC throughout 28 days of storage. These findings are consistent with previous reports showing enhanced extraction efficiency and improved preservation of phenolic compounds in NADES-based lavender extracts of dates and figs compared with conventional extraction methods [37,49]. Based on Pillai’s multivariate test, as shown in Table 3, TPC values are significantly affected by storage time and NADES-based lavender extract addition. While storage time had a negative effect on TPC, NADES-based lavender extract addition showed a positive effect on TPC.

The effect of NADES-based lavender extract supplementation on antioxidant capacity was more evident in date-based formulations than in fig bars. DPPH values exhibited minor fluctuations during storage, which may be associated with the limited diffusion of oxygen and restricted mobility of reactive species within the dense, fibrous structure of fruit bars, able to slow oxidative reactions despite prolonged storage.

After 28 days, differences in DPPH retention among formulations were relatively small but distinguishable at the descriptive level. The D + N formulation exhibited the highest stability (80.48%), corresponding to the smallest reduction in antioxidant capacity. Non-supplemented date (D) and fig (F) bars showed comparable stability values (79.30% and 79.60%, respectively), indicating similar preservation of radical-scavenging activity. In contrast, the F + N sample showed the lowest retention (76.00%), suggesting a greater numerical decrease during storage.

The addition of the NADES-based lavender extract slightly improved DPPH stability in date bars (≈1.2% increase; D → D + N), whereas a small decrease was observed in fig bars (≈3.6%; F → F + N) (Table S1). This indicates that the influence of NADESs on antioxidant stability is matrix-dependent, likely reflecting differences in phenolic composition and interactions between NADES components and the specific fruit matrix. Pillai’s test confirmed a significant effect of storage time—as well as the combined effects of storage time, NADES-based lavender extract, and composition—on the DPPH values (Table 3). The effect of initial ingredient selection and formulation (date, fig, or NADES lavender extract addition) was significant and in agreement with data from the literature [48].

The FRAP assay measures ferric ion-reducing ability and closely follows total phenolic content and other antioxidant indicators. A gradual decline in antioxidant capacity during storage was observed for all fruit bar formulations; however, statistical analysis revealed that the magnitude and timing of this decline depended on formulation composition.

A significant reduction in FRAP values between the initial and final storage points (0 vs. 28 days) was observed for all samples. The decrease was most pronounced in fig bars (F), indicating lower antioxidant stability during storage. In contrast, bars supplemented with the NADES-based lavender extract (D + N and F + N) exhibited higher FRAP retention throughout storage than the corresponding non-supplemented formulations, suggesting improved preservation of antioxidant capacity.

The addition of the NADES-based extract slightly improved FRAP stability in both date and fig bars, reducing the rate of antioxidant loss by approximately 3.7% (D → D + N) and 3.6% (F → F + N). These findings suggest that supplementation with the NADES-based lavender extract was associated with improved preservation of antioxidant capacity during storage (Table S1).

Statistical analysis of the effects of storage time, formulation, and NADES-based lavender extract on the antioxidant capacity determined by the FRAP method showed a significant effect of storage time, composition, and NADES-based lavender extract, as well as their combined effect, on the experimentally determined FRAP values (Table 3).

Furthermore, the microbiological stability of the fruit bars was evaluated on days 0, 15, and 30 of storage, according to standardized ISO microbiological methods. For easier comparison among formulations, the results are presented as the percentage of positive samples in Table 4.

Based on data from the literature, NADESs can exhibit antimicrobial properties, including antibacterial and antifungal activities [50,51]. This paper tested the antimicrobial activity of NADES-based lavender-extract-supplemented fruit bars on bacterial strains (aerobic mesophiles, *Staphyloccocus aureus* and *Salmonela* spp.), as well as yeasts and molds. The initial analysis of the samples immediately after production revealed that none of the microorganisms were present in the fruit bars, making them microbiologically safe and adequate for human consumption. The same trend was maintained after 15 days of storage. After 30 days of storage, differences between supplemented and non-supplemented date-based bars became evident. The date-based bars, both supplemented and non-supplemented, remained stable after 15 days of storage at all temperatures, but after 30 days of storage the effect of the NADES-based lavender extract became evident. Yeasts and molds were detected in 60% of the non-supplemented date bars (D) stored at 22 °C, whereas only 20% of the NADES-supplemented date bars (D + N) stored under the same conditions tested positive. The NADES-supplemented date bars showed a significantly lower percentage of yeast- and mold-positive samples (20%) compared with the non-supplemented date bars (60%). No yeast or mold growth was detected in either fig-based formulation (F and F + N) at any storage temperature or storage time. Furthermore, none of the analyzed bars showed the presence of aerobic mesophilic bacteria, *Enterobacteriaceae*, coagulase-positive *Staphylococcus aureus*, or *Salmonella* spp. throughout the entire storage period. Similar to chemical and sensory properties, the stabilizing effect was much more pronounced with the date-based bars, indicating a high dependence of the level of stabilization on the food matrix and its constituents. Future research on fruit bars should focus on the optimization of the amount of NADES-based extract to be added to the food material, with the aim of achieving satisfactory stability without hindering the sensory acceptance of the products. The level of addition was not the topic of this study, since similar studies, but on a different food matrix (cocoa), have already been published [22].

In general, the addition of lavender NADES extract significantly affected the moisture levels of the bars. NADES-based lavender extract addition raised the moisture content of the bars, lowered their pH, and increased their TPC, DPPH, and FRAP values, thereby enhancing the functional properties of the bars. Furthermore, addition of the NADES-based lavender extract improved TPC stability over 28 days and slightly improved DPPH and FRAP stability in both date and fig bars. The incorporation of the NADES extract markedly improved the microbiological stability of date-based fruit bars by reducing the occurrence of yeast- and mold-positive samples at 22 °C after 30 days of storage, thereby demonstrating a stronger matrix-dependent stabilizing effect compared to non-supplemented samples. Altogether, NADES supplementation enhanced both functional value and storage stability, particularly in date-based matrices, confirming the potential of NADES technology for improving the quality and shelf life of natural fruit bar formulations.

### 3.3. Sensory Properties of Fruit Bars

**Figure 2:**
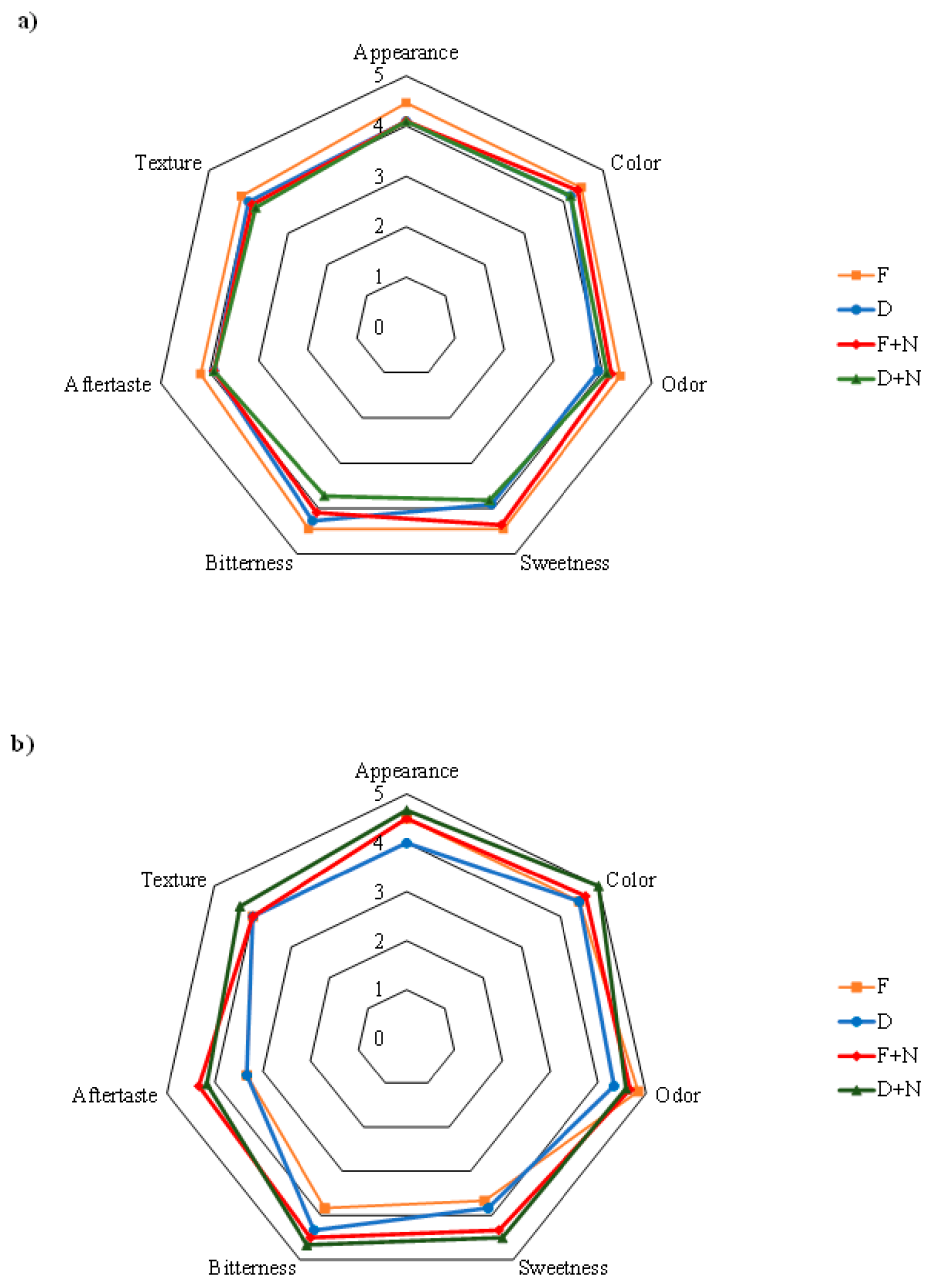
Sensory scores of the samples on day 0 (**a**) and day 28 (**b**) of storage. D—date fruit bar; D + N—lavender–NADES-supplemented date bar; F—fig fruit bar; F + N—lavender–NADES-supplemented fig bar.

Sensory traits of the produced fruit bars included basic tastes of the product, as well as mouthfeel and texture, which were evaluated immediately after production and after 28 days of storage. The results are shown in Figure 2. Also, the influence of storage time, composition, and NADES-based lavender extract on the sensory traits is shown in Table 5.

On day 0 of storage (Figure 2a), the highest scores were obtained by the fig-based fruit bars for all seven traits that were evaluated. This sample was followed by the date-based bars and the lavender–NADES-supplemented fig bars, while the lavender–NADES-supplemented date-based bars proved to be the least acceptable for the panelists. Specific differences can be seen primarily in the scores for odor, sweetness, and bitterness. Specifically, odor was more acceptable for the fig-based bars, equally for the supplemented and the non-supplemented ones, while the date-based bars had lower scores for odor. The same trend was visible for the sweetness trait, where fig-based bars proved to be sweeter than the date-based ones, which was more acceptable for the panelists. In the case of bitterness, the lavender–NADES-supplemented bars, both fig- and date-based, showed lower scores than the non-supplemented ones, which was likely the result of an intensive lavender essential oil flavor, as well as added sourness from the citric acid in the NADES. Panelists preferred the color of the fig-based supplemented and non-supplemented bars, the texture of the fig-based non-supplemented bars, and the appearance of fig-based non-supplemented bars. In comparison to data from the literature, formulations using up to 50–60% date paste in bars generally received the highest sensory scores for texture, flavor, and overall acceptability, as tested by a substantial number of panelists using a 9-point hedonic scale [52]. Furthermore, bars combining figs and dates (e.g., 80:20 ratio) leverage the natural sweetness and nutrition of these fruits and have received positive sensory evaluations from trained panelists [53,54].

After 28 days of storage (Figure 2b), a significant change was detected in the sensory scores and the preferences of the panelists. In all evaluated traits except odor, the lavender–NADES-supplemented fruit bars showed higher scores in comparison to the non-supplemented ones. The lower odor scores observed for the supplemented bars indicate that the aroma profile of the formulation was less acceptable to the panelists. However, the supplemented bars maintained higher sensory scores than the non-supplemented bars after 28 days of storage. The most marked changes were visible in sweetness, bitterness, and aftertaste, where the supplemented bars significantly outscored the non-supplemented ones.

Pillai’s test (Table 5), revealed a significant influence of storage time on appearance, color, and odor. A combined effect of storage time and NADES-based lavender extract addition was apparent for appearance, sweetness, bitterness, and aftertaste, while the composition of the bar was mostly connected to the panelists’ perception of sweetness. These findings further emphasize the importance of optimizing fruit bar formulations, as well as their fortification with NADES-based extracts. The importance of composition optimization and the sensory evaluation of fruit bars was also pointed out in a study by Chaudhary and Chauhan, who concluded that the fruit bars, in addition to a nutritionally beneficial composition, have to be sensorially and price-wise acceptable to the panelists [55].

When comparing the interconnectivity of the sensory properties, it can be seen from Table 5 that the most important properties to which panelists pay attention are appearance, color, odor, sweetness, and texture.

The preserved sensory properties of the fruit bars after 28 days of storage were most pronounced when a lavender extract in a multicomponent NADES was used, suggesting that the extract contributed to stability and reduced oxidation, while components of the NADES (e.g., vitamin C and citric acid) likely further enhanced these protective effects, confirming the potential of NADES-based formulations as functional preservatives in food products. Nevertheless, the odor scores of the lavender-supplemented bars remained relatively lower after storage, indicating that the characteristic lavender aroma may not be equally acceptable to all panelists. Therefore, although NADES supplementation improved the stability of most sensory attributes, further optimization of lavender extract concentration may be required to achieve a better balance between functional benefits and sensory acceptability.

In order to determine whether the addition of lavender NADES extract also affected long-term sensory properties, relative stability was calculated, and the results are presented in Table 6.

When interpreting the results, it is important to note that the negative values represent a decline in the analyzed property, while the positive ones represent an improvement.

The most pronounced changes in the stability of the fruit-based bars with the addition of lavender NADES extract were seen with respect to sensory properties. Lavender NADES extract was associated with better preservation of sensory scores for appearance, color, odor, sweetness, aftertaste, and texture. The most affected properties by far were sweetness and aftertaste, where the degradation with storage time was markedly higher for the non-supplemented bars, while the bars that contained the extract received even higher grades after 28 days of storage. Although there was no marked difference in the texture of the bars at the beginning and the end of storage, the addition of the lavender NADES extract increased the smooth mouthfeel of the bars, which led to higher grades for texture after 28 days of storage.

## 4. Conclusions

This research evaluated the impact of NADES-based lavender extract supplementation on the physicochemical, sensory, microbiological, and stability-related properties of fruit bars formulated from dates or figs and 33.58% grape pomace. The formulation combined the delivery of functional bioactive compounds with the valorization of grape pomace—a major winery by-product that is often underutilized despite its valuable nutritional and phytochemical composition—thereby supporting sustainable food production and circular economy principles. The combined multicomponent NADES-based lavender extract was successfully incorporated into upcycled fruit bars and was associated with increased total phenolic content, enhanced antioxidant capacity, higher moisture content, lower pH, and improved sensory attributes after 28 days of storage. The formulation was also associated with lower yeast and mold counts in date-based fruit bars after 28 days of storage, as well as with the maintenance of several sensory attributes, including appearance, color, odor, sweetness, bitterness, and aftertaste. However, since a blank NADES control was not included in the final experimental design, the individual contributions of the NADES matrix and lavender-derived compounds could not be distinguished. Therefore, the observed effects should be attributed to the combined NADES–lavender extract formulation. The obtained results indicate that the combined NADES–lavender extract formulation shows potential as a functional ingredient in fruit bars, particularly in date-based formulations. Further studies including blank NADES controls, longer storage periods, sensory acceptability, and safety evaluation of the final formulation are required before commercial application. Future studies should also investigate food-grade NADES formulations without plant extracts, in order to determine whether the NADES matrix itself possesses intrinsic antioxidant, antimicrobial, or stabilizing properties, and to clarify its individual contribution to food quality.

## Figures and Tables

**Table 1:** Individual phenolic compounds, total polyphenol content (TPC), and antioxidant activity (FRAP and DPPH) of the lavender NADES extract.

Compound	Content
Gallic acid	0.3 ± 0.0
Catechin	1.7 ± 0.4
Syringic acid	0.7 ± 0.9
Chlorogenic acid	3.2 ± 0.0
Trans-sinapic acid	0.7 ± 0.2
Luteolin-7-o-glucoside	0.3 ± 0.0
Rosmarinic acid	5.3 ± 0.4
Quercetin	1.3 ± 0.2
TPC	15.0 ± 0.1
DPPH	1.5 ± 0.1
FRAP	5.5 ± 0.1

Values are expressed as the mean ± SD (*n* = 3). Individual phenolic compounds are expressed as μg g^−1^ dry weight (dw) of lavender, TPC as mg GAE g^−1^ dw, FRAP as mmol FeSO_4_·7H_2_O g^−1^ dw, and DPPH as mmol Trolox g^−1^ dw.

**Table 2:** Dry matter, pH, total dissolved solids, and conductivity of the fruit bars.

Property	Dry Matter Content (%) *	pH (Extract) **	Total Dissolved Solids (mg L^−1^) **	Conductivity (µS cm^−1^) **
Date fruit bar				
Day 0	64.36 ± 1.34 ^a^	5.55 ± 0.03 ^a^	61.83 ± 1.00 ^a^	129.87 ± 1.95 ^a^
Day 7	62.76 ± 0.16 ^b^	5.59 ± 0.01 ^b^	55.73 ± 1.35 ^b^	121.70 ± 1.85 ^b^
Day 14	62.64 ± 0.03 ^c^	5.64 ± 0.02 ^c^	78.20 ± 1.31 ^c^	164.00 ± 2.55 ^c^
Day 21	63.19 ± 0.12 ^c^	5.60 ± 0.01 ^d^	78.53 ± 1.10 ^d^	165.49 ± 1.00 ^d^
Day 28	61.41 ± 0.36 ^b^	5.60 ± 0.01 ^d^	78.20 ± 1.30 ^c^	164.00 ± 2.55 ^c^
Date + lavender NADES fruit bar				
Day 0	63.45 ± 0.47 ^a^	5.49 ± 0.01 ^a^	62.67 ± 0.40 ^a^	119.30 ± 4.30 ^a^
Day 7	61.28 ± 0.15 ^b^	5.48 ± 0.01 ^b^	66.23 ± 0.45 ^b^	128.50 ± 3.12 ^b^
Day 14	61.85 ± 0.44 ^b^	5.51 ± 0.02 ^c^	83.37 ± 0.78 ^c^	169.00 ± 0.89 ^c^
Day 21	63.03 ± 0.40 ^a^	5.52 ± 0.03 ^d^	80.20 ± 0.30 ^d^	158.33 ± 1.16 ^d^
Day 28	63.15 ± 0.12 ^a^	5.36 ± 0.06 ^e^	80.43 ± 0.57 ^e^	167.07 ± 0.61 ^e^
Fig fruit bar				
Day 0	61.04 ± 2.17 ^a^	5.59 ± 0.07 ^a^	46.37 ± 3.26 ^a^	93.97 ± 0.58 ^a^
Day 7	62.18 ± 0.17 ^b^	5.56 ± 0.00 ^b^	53.93 ± 1.40 ^b^	115.87 ± 1.90 ^b^
Day 14	59.62 ± 0.01 ^c^	5.56 ± 0.03 ^b^	69.83 ± 1.03 ^c^	144.03 ± 1.20 ^c^
Day 21	61.80 ± 0.46 ^a^	5.57 ± 0.03 ^c^	67.43 ± 0.49 ^d^	155.20 ± 1.57 ^d^
Day 28	63.07 ± 0.26 ^e^	5.55 ± 0.04 ^d^	69.83 ± 1.02 ^c^	144.03 ± 1.20 ^c^
Fig + lavender NADES fruit bar				
Day 0	64.13 ± 0.27 ^a^	5.49 ± 0.02 ^a^	52.47 ± 0.40 ^a^	101.50 ± 1.31 ^a^
Day 7	63.04 ± 0.38 ^b^	5.43 ± 0.01 ^b^	52.90 ± 0.56 ^b^	101.43 ± 1.02 ^a^
Day 14	63.23 ± 0.15 ^c^	5.45 ± 0.01 ^c^	58.03 ± 0.45 ^c^	102.23 ± 1.25 ^b^
Day 21	63.93 ± 0.21 ^d^	5.43 ± 0.09 ^b^	61.77 ± 0.71 ^d^	117.40 ± 2.51 ^c^
Day 28	63.25 ± 0.81 ^c^	5.40 ± 0.06 ^d^	68.33 ± 0.45 ^c^	132.23 ± 1.25 ^d^

* Determined in the fruit bar. ** Determined in the fruit bar extract. Different letters in the same column for the same fruit bar type represent significant differences determined by the Dunn–Bonferroni post hoc test at *p* < 0.05.

**Table 3:** Multivariate Pillai’s test results for estimation of mixed effects on physical and chemical properties of the fruit bars. Values marked bold are significant at *p* < 0.05.

Effect/Property	Pillai’s Trace	F	Effect df	Error df	*p*
Dry matter content					
**Storage**	**0.959519**	**29.62870**	**4**	**5**	**0.001121**
Storage * Composition	**0.980375**	**62.44309**	**4**	**5**	**0.000186**
Storage * NADES	**0.977204**	**53.58339**	**4**	**5**	**0.000270**
Storage * Composition * NADES	**0.984430**	**79.03143**	**4**	**5**	**0.000105**
pH					
Storage	0.682804	2.690783	4	5	0.153394
Storage * Composition	0.668777	2.523888	4	5	0.168706
Storage* NADES	0.389743	0.798316	4	5	0.574392
Storage * Composition * NADES	0.419949	0.904984	4	5	0.525281
Total dissolved solids					
**Storage**	**0.999909**	**13672.19**	**4**	**5**	**0.000000**
Storage * Composition	**0.997150**	**437.40**	**4**	**5**	**0.000002**
Storage * NADES	**0.996805**	**389.98**	**4**	**5**	**0.000002**
Storage * Composition* NADES	**0.996877**	**399.04**	**4**	**5**	**0.000002**
Conductivity					
**Storage**	**0.999919**	**15470.81**	**4**	**5**	**0.000000**
Storage * Composition	**0.998906**	**1141.58**	**4**	**5**	**0.000000**
Storage * NADES	**0.997836**	**576.27**	**4**	**5**	**0.000001**
Storage * Composition* NADES	**0.999425**	**2172.85**	**4**	**5**	**0.000000**
TPC					
**Storage**	**0.983287**	**73.54222**	**4**	**5**	**0.000125**
Storage * Composition	0.555753	1.56375	4	5	0.314301
Storage * NADES	**0.917791**	**13.95523**	**4**	**5**	**0.006384**
Storage * Composition * NADES	0.800359	5.01125	4	5	0.053441
DPPH					
**Storage**	**0.999921**	**15863.23**	**4**	**5**	**0.000000**
Storage * Composition	0.768039	4.14	4	5	0.075672
Storage * NADES	**0.999769**	**5417.62**	**4**	**5**	**0.000000**
Storage * Composition* NADES	**0.997837**	**576.56**	**4**	**5**	**0.000001**
FRAP					
**Storage**	**0.995569**	**280.8801**	**4**	**5**	**0.000005**
Storage * Composition	**0.930831**	**16.8217**	**4**	**5**	**0.004186**
Storage * NADES	**0.958130**	**28.6044**	**4**	**5**	**0.001218**
Storage * Composition* NADES	**0.977027**	**53.1617**	**4**	**5**	**0.000275**

**Table 4:** Microbiological stability of fruit bars during storage.

Storage Day	D + N	D	F + N	F
0	0	0	0	0
15	0 (4 °C) 0 (22 °C)	0 (4 °C) 0 (22 °C)	0 (4 °C) 0 (22 °C)	0 (4 °C) 0 (22 °C)
30	0 (4 °C) 1 in 5 samples (20%) positive on yeasts and moulds (22 °C)	0 (4 °C) 3 in 5 samples (60%) positive on yeasts and moulds (22 °C)	0 (4 °C) 0 (22 °C)	0 (4 °C) 0 (22 °C)

D—date fruit bar; D + N—lavender–NADES-supplemented date bar; F—fig fruit bar; F + N—lavender–NADES-supplemented fig bar.

**Table 5:** Pillai’s univariate test results for the estimation of the effects of storage time, composition, and NADES-based lavender extract on the sensory properties of fruit bars. Values marked in bold are significant at *p* < 0.05.

Effect/Property	SS	Degrees of Freedom	MS	F	*p*
Appearance					
**Storage**	**1.32369**	**1**	**1.323691**	**4.280624**	**0.044455**
Storage * Composition	0.00138	1	0.001377	0.004454	0.947090
Storage * NADES	**1.59229**	**1**	**1.592287**	**5.149220**	**0.028213**
Storage * Composition * NADES	0.13774	1	0.137741	0.445434	0.507997
Color					
**Storage**	**3.30716**	**1**	**3.307163**	**8.307958**	**0.006083**
Storage * Composition	0.93113	1	0.931129	2.339100	0.133321
Storage*NADES	0.86088	1	0.860882	2.162630	0.148518
Storage * Composition* NADES	0.08815	1	0.088154	0.221453	0.640259
Odor					
**Storage**	**5.25215**	**1**	**5.252152**	**13.98327**	**0.000530**
Storage * Composition	0.00215	1	0.002152	0.00573	0.940003
Storage * NADES	0.01042	1	0.010417	0.02773	0.868501
Storage * Composition * NADES	0.00422	1	0.004218	0.01123	0.916083
Sweetness					
Storage	0.06749	1	0.067493	0.113426	0.737878
Storage * Composition	**3.04270**	**1**	**3.042700**	**5.113426**	**0.028736**
Storage * NADES	**3.44353**	**1**	**3.443526**	**5.787037**	**0.020415**
Storage * Composition * NADES	0.00000	1	0.000000	0.000000	1.000000
Bitterness					
Storage	0.93113	1	0.931129	1.052960	0.310435
Storage * Composition	2.20386	1	2.203857	2.492212	0.121574
Storage* NADES	**5.46694**	**1**	**5.466942**	**6.182243**	**0.016772**
Storage * Composition * NADES	0.03444	1	0.034435	0.038941	0.844475
Aftertaste					
Storage	0.82679	1	0.826791	1.226878	0.274034
Storage * Composition	0.01687	1	0.016873	0.025038	0.874996
Storage * NADES	**6.65324**	**1**	**6.653237**	**9.872764**	**0.002999**
Storage * Composition * NADES	0.28960	1	0.289601	0.429739	0.515530
FRAP	0.82679	1	0.826791	1.226878	0.274034
Texture					
Storage	0.26997	1	0.269972	0.521277	0.474117
Storage * Composition	0.55096	1	0.550964	1.063830	0.307980
Storage * NADES	0.93113	1	0.931129	1.797872	0.186851
Storage * Composition * NADES	0.08815	1	0.088154	0.170213	0.681926

**Table 6:** Relative stability (%) of sensory properties of the fruit bar samples after 28 days of storage.

	Relative Stability (%)			
Sample	D + N	D	F + N	F
Appearance	14.07	−2.22	10	1.02
Color	19.56	7.61	6.94	1.02
Odor	12.04	10.85	11.59	10.76
Sweetness	17.86	−1.94	−0.69	−17.69
Bitterness	25.20	1.42	10	−13.95
Aftertaste	6.59	−14.73	10.85	−20.29
Texture	13.49	0	2.32	−4.35

## Data Availability

The original contributions presented in this study are included in the article/Supplementary Material. Further inquiries can be directed to the corresponding author.

## Supplementary Materials

The following supporting information can be downloaded at https://www.mdpi.com/article/10.3390/molecules31152608/s1: Figure S1. Representative HPLC-DAD chromatogram of the lavender NADES extract. Table S1. Relative changes in TPC, DPPH and FRAP stability after 28 days of storage.
