# Supplementary material for: Enhancing Antioxidant and Storage Stability of Upcycled Fruit Bars Through NADES-Based Lavender Extract Addition
Source: Molecules. 2026 Jul 27;31(15):2608. doi: 10.3390/molecules31152608 (PMC13468640; doi:10.3390/molecules31152608)

Figure S1. Representative HPLC-DAD chromatogram of the lavender NADES extract.

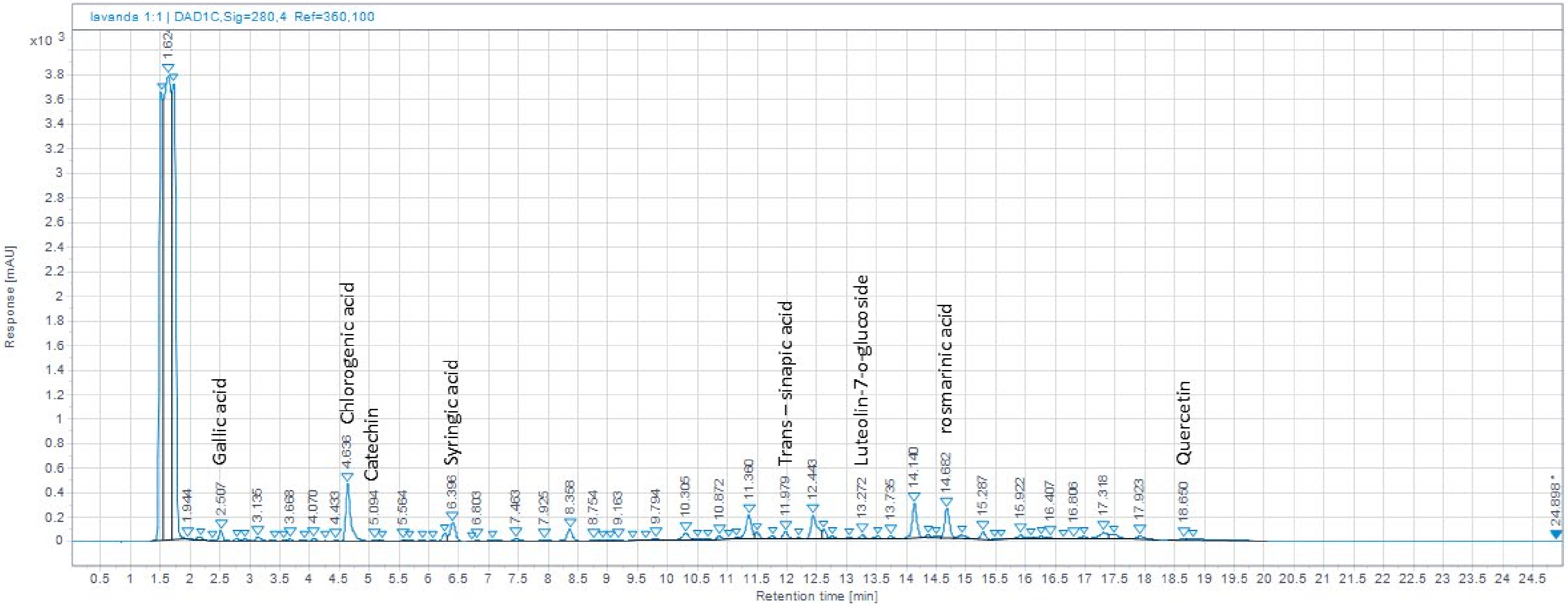

Supplement: Supplementary file 1 [file molecules-31-02608-s001.zip › Figure S1.pdf]
