# Supplementary material for: Enhancing Antioxidant and Storage Stability of Upcycled Fruit Bars Through NADES-Based Lavender Extract Addition
Source: Molecules. 2026 Jul 27;31(15):2608. doi: 10.3390/molecules31152608 (PMC13468640; doi:10.3390/molecules31152608)
Supplement: Supplementary file 1 [file molecules-31-02608-s001.zip › Table S1.pdf]

Table S1. Relative changes in TPC, DPPH and FRAP stability after 28 days of storage

Parameter	Fig bars (F → F+N)	Date bars (D → D+N)
TPC (%)	+2.8	+10.3
DPPH (%)	−3.6	+1.2
FRAP (%)	+3.6	+3.7

D - date fruit bar; D+N - lavender NADES supplemented date bar; F - fig fruit bar; F+N - lavender NADES supplemented fig bar
